# Progress Toward Hepatitis B Control — South-East Asia Region, 2016–2019

**DOI:** 10.15585/mmwr.mm6930a2

**Published:** 2020-07-31

**Authors:** Hardeep S. Sandhu, Sigrun Roesel, Mohammad Sharifuzzaman, Supamit Chunsuttiwat, Rania A. Tohme

**Affiliations:** ^1^Global Immunization Division, Center for Global Health, CDC; ^2^South-East Asia Regional Office, Immunization and Vaccine Development, World Health Organization, New Delhi, India; ^3^Department of Disease Control, Ministry of Public Health, Royal Thai Government, Nonthaburi, Thailand.

In 2015, the World Health Organization (WHO) South-East Asia Region (SEAR)* reported an estimated 40 million persons living with chronic hepatitis B virus (HBV) infection and 285,000 deaths from complications of chronic infection, cirrhosis, and hepatocellular carcinoma (*1*). Most chronic HBV infections, indicated by the presence of hepatitis B surface antigen (HBsAg) on serologic testing, are acquired in infancy through perinatal or early childhood transmission (*2*). To prevent perinatal and childhood infections, WHO recommends that all infants receive at least 3 doses of hepatitis B vaccine (HepB), including a timely birth dose (HepB-BD)^†^ (*1*). In 2016, the SEAR Immunization Technical Advisory Group endorsed a regional hepatitis B control goal with a target of achieving hepatitis B surface antigen (HBsAg) seroprevalence of ≤1% among children aged ≥5 years by 2020, which is in line with the WHO Global Health Sector Strategy on Viral Hepatitis 2016–2021 (*2*,*3*). The South-East Asia Regional Vaccine Action Plan 2016–2020 (SEARVAP) (*4*) identified the acceleration of hepatitis B control as one of the eight regional goals for immunization. The plan outlined four main strategies for achieving hepatitis B control: 1) achieving ≥90% coverage with 3 doses of HepB (HepB3), 2) providing timely vaccination with a HepB birth dose (HepB-BD), 3) providing catch-up vaccination of older children, and 4) vaccinating adult populations at high risk and health care workers (*1*,*4*). In 2019, SEAR established a regional expert panel on hepatitis B to assess countries’ HBV control status. This report describes the progress made toward hepatitis B control in SEAR during 2016–2019. By 2016, all 11 countries in the region had introduced HepB in their national immunization programs, and eight countries had introduced HepB-BD. During 2016–2019, regional HepB3 coverage increased from 89% to 91%, and HepB-BD coverage increased from 34% to 54%. In 2019, nine countries in the region achieved ≥90% HepB3 coverage, and three of the eight countries that provide HepB-BD achieved ≥90% HepB-BD coverage. By December 2019, four countries had been verified to have achieved the hepatitis B control goal. Countries in the region can make further progress toward hepatitis B control by using proven strategies to improve HepB-BD and HepB3 coverage rates. Conducting nationally representative hepatitis B serosurveys among children will be key to tracking and verifying the regional control targets.

## Immunization Activities

HepB-BD and HepB3 coverage data are reported annually to WHO and the United Nations Children’s Fund (UNICEF) from all 11 SEAR countries. WHO and UNICEF use country-reported survey and administrative coverage data (number of vaccine doses administered divided by the estimated target population) to estimate vaccination coverage. By 2016, all countries in the region had introduced at least 3 HepB doses into national immunization schedules, and eight countries had introduced universal HepB-BD vaccination in addition to HepB3 (Table 1) (*5*). Since 1992, Thailand has provided 4 doses of HepB (at ages 0, 2, 4, and 6 months) for all infants and administers an extra dose at age 1 month for infants born to mothers with positive test results for HBsAg (*6*). During 2016–2019, regional HepB3 coverage increased from 89% to 91%. By 2019, nine countries had reached the regional target of ≥90% HepB3 coverage, six had reached ≥95% HepB3 coverage, and four countries reported HepB3 coverage of ≥80% in all districts^§^ (Table 1). Regional HepB-BD coverage increased from 34% in 2016 to 54% in 2019. Three of the eight countries that had introduced HepB-BD achieved HepB-BD coverage of ≥90% in 2019. HepB-BD coverage in India, the country with the largest birth cohort in the region, was <60% during 2016–2019 (*5*).

**TABLE 1:** Hepatitis B vaccine (HepB) schedule and estimated coverage* with a birth dose and third dose of HepB, by country — World Health Organization (WHO) South-East Asia Region, 2016–2019

Country/Area	No. of live births, 2019	HepB schedule	Year HepB introduced	Year birth dose introduced	% Coverage					
					2016			2019		
					HepB-BD	HepB3	Districts^†^ with ≥80% HepB3 coverage (%)	Timely HepB-BD^§^	HepB3	Districts^†^ with ≥80% HepB3 coverage (%)
Bangladesh	3,408,614	6, 10, 14 wks	2003	ND	NA	98	100	NA	98	98
Bhutan	11,496	0, 6, 10, 14 wks	1997	2012	82	98	100	86	97	100
Burma^¶^	981,223	0, 2, 4, 6 mos	2003	2016	NA	90	88	17	90	84
India	27,192,790	0, 6, 10, 14 wks	2002^¶^	2011	47	88	69	56	91	77
Indonesia	4,766,582	0, 2, 3, 4, 18 mos	1997	2002	NA	84	74	84	85	77
Maldives	5,964	0, 2, 4, 6 mos	1993	2000	NA	99	100	99	99	100
Nepal	640,789	6, 10, 14 wks	2002	ND	NA	87	68	NA	93	69
North Korea	325,605	0, 6, 10, 14 wks	2003	2004	98	96	100	98	97	100
Sri Lanka	329,754	2, 4, 6 mos	2003	ND	NA	99	100	NA	99	100
Thailand	600,267	0, 2, 4, 6 mos**	1992	1992	NA	99	NR	99	97	95
Timor-Leste	47,269	0, 6, 10, 14 wks	2007	2016	42	79	100	70	83	54
**South-East Asia Region**	**38,314,010**	**—**	**—**	**—**	**34**	**89**	**—**	**54**	**91**	**—**
**Global**	**139,677,000**	**—**	**—**	**—**	**35**	**84**	**—**	**43**	**85**	**—**

**Abbreviations:** HepB-BD = birth dose of monovalent hepatitis B vaccine; HepB3 = third dose of hepatitis B containing vaccine; mos = months; NA = not applicable; ND = not done; NR = not reported; UNICEF = United Nations Children’s Fund; wks = weeks.

* WHO-United Nations Children’s Fund estimates. https://www.who.int/immunization/monitoring_surveillance/data/en/.

^†^ For Maldives and Thailand, district-level HepB3 coverage data are provided for province and atolls only, respectively.

^§^ Timely hepatitis B birth-dose is defined as administration of a dose of hepatitis B vaccine within 24 hours of birth.

^¶^ HepB introduction was piloted in 2002 and made universal in 2011. https://extranet.who.int/iris/restricted/bitstream/handle/10665/329981/India2019_epi-eng.pdf?sequence=1&isAllowed=y.

** An additional HepB dose given at 1 month for infants born to a mother chronically infected with hepatitis B virus, in addition to birth dose and routine infant doses.

## HBsAg Seroprevalence Surveys

HBV infections in children are typically asymptomatic, but can lead to liver cirrhosis and cancer in adulthood. Therefore, to assess the effectiveness of the hepatitis B immunization program in preventing HBV infections, nationally representative surveys are conducted to determine HBsAg seroprevalence among children aged ≥5 years. Measuring HBsAg prevalence among children aged ≥5 years accounts for the period of highest risk for perinatal or horizontal transmission of HBV and of becoming chronically infected with HBV (*2*). During 2011–2017, seroprevalence studies were conducted in six countries: Bangladesh, Bhutan, Burma, Indonesia, Nepal, and Thailand. HBsAg seroprevalence before vaccine introduction ranged from 0.3% to 7% (Table 2). In four (Bangladesh, Bhutan, Nepal, and Thailand) of five countries where seroprevalence data were collected after vaccine introduction, HBsAg prevalence declined to <1%.

**TABLE 2:** Hepatitis B surface antigen (HBsAg) seropositivity, by country — World Health Organization South-East Asia Region, 2011–2017

Country	Year of most recent representative HBsAg seroprevalence survey	No. of persons tested	HBsAg seroprevalence, before vaccine introduction % (95% CI)	HBsAg seroprevalence in children aged ≥5 years,* after vaccine introduction % (95% CI)	Year of verification of ≤1% HBsAg seroprevalence^†^
Bangladesh^§^	2011–2012	2,100 prevaccine; 2,100 postvaccine	1.2 (0.7–1.6)	0.05 (0.0–0.1)	2019
Bhutan^¶^	2017	775 prevaccine; 546 postvaccine	2 (1.0–4.0)	0.5 (0.1–1.8)	2019
Burma**	2015	5,547 prevaccine only^††^	6.5 (5.9–7.2)	ND	NS
India	ND	—	—	—	NS
Indonesia^§§^	2013	Total sample of >15,000^§§^	7 (NR)	4.20 (NR)	NS
Maldives	ND	—	—	—	NS
Nepal^¶¶^	2012	1,200 prevaccine; 2,186 postvaccine	0.3 (0.1–0.9)	0.1 (0.04–0.4)	2019
North Korea	ND	—	—	—	NS
Sri Lanka	ND	—	—	—	NS
Thailand***	2014	2,805 prevaccine; 3,159 postvaccine^§§^	4.5 (NR)	0.3 (NR)	2019
Timor-Leste	ND	—	—	—	NS

**Abbreviations:** CI = confidence interval; ND = not done; NR = not reported; NS = not submitted to the regional verification commission.

* Born after the nationwide implementation of universal hepatitis B infant immunization.

^†^ Verification is done by a regional commission of experts from the Hepatitis B immunization Expert Resource Panel that determines if the country has reached the target of ≤1% HBsAg seroprevalence among children aged 5 years.

^§^ http://www.ajtmh.org/content/journals/10.4269/ajtmh.17-0721.

^¶^ World Health Organization. Serosurvey to estimate the prevalence of biomarkers of infections with hepatitis B and C viruses, and antibodies to measles and rubella Bhutan, March–April 2017. New Delhi, India: World Health Organization, Regional Office for South-East Asia Office; 2017.

** Lwin AA, Aye KS, Htun MM, et al. Seroprevalence of hepatitis B and C viral infections in Myanmar: national and regional survey in 2015. Myanmar Health Sci Res J 2017;29(3).

^††^ Prevaccine sample included adults.

^§§^ Muljono DH. Epidemiology of hepatitis B and C in Republic of Indonesia. Eurasian J Hepato-Gastroenterol 2017;7:59-9.

^¶¶^ https://doi.org/10.1016/j.vaccine.2014.06.027.

*** https://doi.org/10.1371/journal.pone.0150499.

## Regional Verification of Hepatitis B Control Goal

In 2019, the WHO SEAR Office established the South-East Asia Regional Expert Panel (SEA REP), consisting of eight regional and international independent experts in hepatitis B, immunization, hepatology, and epidemiology, to verify each country’s status in achieving the regional hepatitis B control goal through immunization.^¶^ SEA REP established two essential criteria for verifying hepatitis B control achievement: 1) a nationally representative seroprevalence survey that documents HBsAg seroprevalence ≤1% among children aged ≥5 years who were born after implementation of nationwide universal hepatitis B infant immunization and 2) coverage with HepB-BD (in countries where HepB-BD is in the national immunization schedule) and HepB3 of ≥90% at national and ≥80% at subnational levels for the previous 5 years, to follow the SEARVAP targets (*1*,*4*). Additional supplementary information may be submitted if available, such as screening of pregnant women for HBsAg during antenatal care, prophylaxis for infants born to mothers with positive test results for HBsAg,** and surveillance for acute hepatitis to guide vaccination strategies among adult populations at high risk. In 2019, SEA REP verified that Bangladesh, Bhutan, Nepal, and Thailand had achieved the regional hepatitis B control target (Table 2) (Figure).

**FIGURE:**
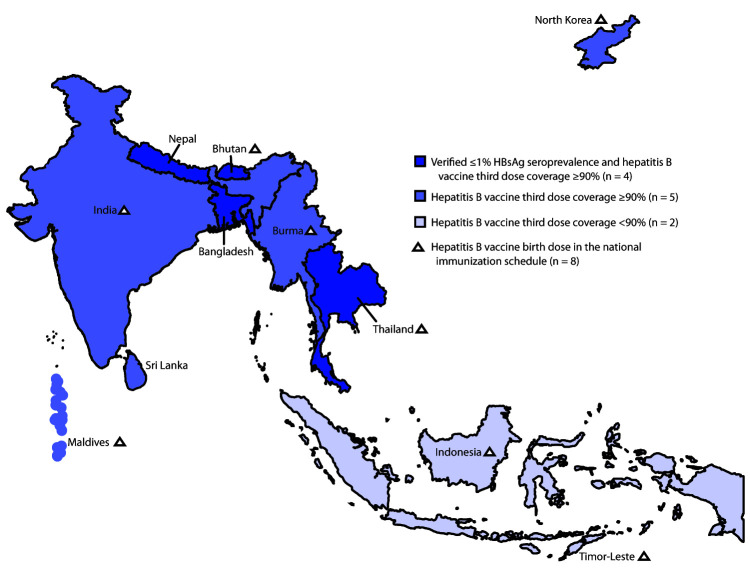
Estimated coverage* with third dose of hepatitis B vaccine and verification of hepatitis B control,^†^ by country —World Health Organization (WHO) South-East Asia Region, 2019 **Abbreviation:** HBsAg = hepatitis B surface antigen. * WHO-United Nations Children’s Fund estimates. https://www.who.int/southeastasia/health-topics/immunization. ^†^ Verification by South-East Asia Regional Expert Panel that determines if the country has reached the target of ≤1% HBsAg seroprevalence among children aged ≥5 years and coverage of third dose of hepatitis B vaccine to be ≥90% at national and ≥80% at subnational levels for the previous 5 years.

## Discussion

During 2016–2019, SEAR made significant progress toward hepatitis B control. HepB has been introduced in all 11 countries in the region and HepB-BD in eight of those countries. By 2019, HepB3 coverage exceeded 90% in all countries except Indonesia and Timor-Leste, and HepB-BD coverage had increased by 59%. By 2019, four countries in the region were verified to have achieved the 2020 regional control target. This progress was substantiated by a hepatitis B modeling study, which estimated that hepatitis B immunization prevented approximately 16 million chronic HBV infections and averted 2.5 million deaths that would have occurred during the lifetime of children born during 1992–2015 (*7*).

Achieving HepB3 coverage of ≥90% nationally and ≥80% in all districts will be essential to achieving hepatitis B control by 2020. However, in India and Indonesia, whose combined birth cohorts account for 83% of SEAR births, <80% of the districts achieved HepB3 coverage of ≥80%, despite intensified vaccination activities targeted at districts with low coverage (*8*). In Nepal, national coverage was ≥90%; however, only 69% of the districts achieved ≥80% HepB3 coverage. Additional strategies that have been successful at improving HepB3 coverage in other countries include 1) implementing online vaccination registration, 2) mapping high-risk areas to identify children who missed doses, 3) verifying complete vaccination on school entry, 4) involving the private sector by providing free vaccines to providers, and 5) addressing vaccine hesitancy through enhanced communication and social mobilization. Including such strategies could help the region accelerate progress toward hepatitis B control (*8*). National coverage inequities could be reduced by conducting catch-up vaccination activities to reach the unvaccinated and increase HepB3 coverage in all districts to ≥80%.

Improving timely HepB-BD coverage is also essential for preventing perinatal transmission of HBV from mother to child and horizontal transmission during early childhood from household members and close contacts. Promoting newborn delivery in health facilities has been shown to increase timely HepB-BD coverage when accompanied by health care worker training, availability of HepB-BD in delivery wards, standing orders for HepB-BD administration, and the presence of skilled birth attendants (*9*). Almost 80% of births in India occur in health facilities, but many births are not assisted by skilled birth attendants (*9*), and timely HepB-BD coverage in 2019 was only 56%. To reach infants born outside health facilities, Indonesia and Timor-Leste instituted national policies allowing use of a compact, prefilled, auto-disable injection device (CPAD) that makes it easier for midwives and traditional birth attendants to administer HepB-BD (*7*,*10*). Indonesia also uses CPAD outside the cold chain for HepB-BD delivery in hard to reach areas, enabling vaccinations for home births in areas lacking cold chain for vaccine storage (*7*).^††^ In India, use of an open vial policy^§§^ to reduce wastage of monovalent HepB vaccine contributed to improvement in HepB-BD coverage.^¶¶^ Educating mothers during prenatal care visits about the importance of a timely HepB-BD and integrating HepB-BD vaccination with essential maternal and newborn care have been shown to increase timely HepB-BD administration, especially in home births in remote, hard-to-reach areas (*9*). Reports from community health workers to health facility personnel about recent births can also help increase timely HepB-BD administration (*9*).

Nationally representative HBsAg serosurveys among children are required to verify achievement of the regional hepatitis B control goal. With sustained national HepB3 coverage of ≥90% and all districts achieving HepB3 ≥80%, Maldives, North Korea, and Sri Lanka only need to conduct serosurveys to determine whether they have reached the control target. Assessing current HBsAg prevalence in India and Indonesia would guide interventions to improve HepB vaccination in specific areas to achieve hepatitis B control.

For some countries that do not provide routine HepB-BD, national serosurvey data might show low seroprevalence. In such countries, screening pregnant women for HBsAg and providing HepB-BD and hepatitis B immunoglobulin to exposed infants would prevent perinatal infections, a key recommendation in the SEARVAP. Establishing perinatal hepatitis B databases to track screening, timely HepB-BD administration, completion of vaccination among exposed newborns, and provision of antiviral treatment to eligible pregnant women would further help prevent mother-to-child transmission of HBV. Close collaboration between the immunization, maternal, neonatal, and child health and viral hepatitis programs are needed to achieve hepatitis B control and elimination.

The findings in this report are subject to at least two limitations. First, estimates of the target population might be inaccurate, resulting in inaccurate vaccination coverage estimates and inaccurate assessments of achievement of the vaccination coverage target. Second, lack of representativeness of some serosurveys and lower sensitivity of the rapid HBsAg test in the field could bias the findings used to determine achievement and validation of hepatitis B control in some countries.

Despite progress in hepatitis B vaccination and verification that four countries have achieved the 2020 control goal, Burma, India, Indonesia, and Timor-Leste are unlikely to achieve hepatitis B control by the end of 2020. Because of the coronavirus disease 2019 pandemic, childhood vaccination coverage rates are declining globally. Interventions to maintain or improve HepB vaccination coverage, particularly HepB-BD, along with other childhood vaccines, will reduce missed opportunities for vaccination and speed progress toward the regional goal.

**Summary**

**What is already known about this topic?**

In 2015, an estimated 40 million persons in the World Health Organization South-East Asia Region had chronic hepatitis B virus infection.

**What is added by this report?**

During 2016–2019, regional hepatitis B vaccine (HepB) birth dose (HepB BD) and third dose (HepB3) coverage increased from 34% to 54% and from 89% to 91%, respectively. In 2019, nine of 11 countries in the region achieved ≥90% HepB3 coverage nationally, and three of eight countries that provide HepB-BD achieved ≥90% HepB-BD coverage. By 2019, four countries achieved hepatitis B control.

**What are the implications for public health practice?**

Enhanced coordination among maternal, newborn, and child health services and immunization services could improve coverage and support achievement of hepatitis B control.

## Footnotes

* The South-East Asia Region, one of the six regions of World Health Organization, consists of 11 countries with a total population of approximately 2 billion, including Bangladesh, Bhutan, Burma, India, Indonesia, Maldives, Nepal, North Korea, Sri Lanka, Thailand, and Timor-Leste.

† Timely hepatitis B birth-dose is defined as administration of a dose of hepatitis B vaccine within 24 hours of birth.

§ Data for Maldives and Thailand for percent district ≥80% HepB3 coverage only for provinces and atolls, respectively.

¶ https://www.who.int/docs/default-source/searo/ivd/guidelines-for-verification-of-achievement-of-hepatitis-b-control-target-through-immunization-in-the-who-sear.pdf.

** Countries that have not introduced HepB-BD recommended to provide evidence of high coverage for antenatal screening for HBV and HepB-BD among infants born to mothers with positive test results for HBsAg.

†† https://www.sciencedirect.com/science/article/pii/S0264410X9900242X?via%3Dihub.

¶¶ All opened WHO-prequalified multidose vials of vaccines should be discarded at the end of the immunization session, or within 6 hours of opening, whichever comes first, unless the vaccine meets all four of the following criteria, in which case, the opened vial can be kept and used for up to 28 days after opening: 1) the vaccine is currently prequalified by WHO; 2) the vaccine is approved for use for up to 28 days after opening the vial, as determined by WHO; 3) the expiry date of the vaccine has not passed; and 4) the vaccine vial has been, and will continue to be, stored at WHO- or manufacturer-recommended temperatures; furthermore, the vaccine vial monitor, if one is attached, is visible on the vaccine label and is not past its discard point, and the vaccine has not been damaged by freezing. https://apps.who.int/iris/bitstream/handle/10665/135972/WHO_IVB_14.07_eng.pdf;sequence=1.
